# Co-electrospun nanofibrous mats loaded with bitter gourd (*Momordica charantia*) extract as the wound dressing materials: in vitro and in vivo study

**DOI:** 10.1186/s12906-021-03284-4

**Published:** 2021-04-07

**Authors:** Mohammad Saeid Salami, Gholamreza Bahrami, Elham Arkan, Zhila Izadi, Shahram Miraghaee, Hadi Samadian

**Affiliations:** 1 Student Research Committee, Kermanshah University of Medical Sciences, Kermanshah, Iran; 2 Medical Biology Research Center, Health Technology Institute, Kermanshah University of Medical Sciences, Kermanshah, Iran; 3 Pharmaceutical Sciences Research Center, Health Institute, Kermanshah University of Medical Sciences, Kermanshah, Iran; 4 Nano Drug Delivery Research Center, Health Technology Institute, Kermanshah University of Medical Sciences, Kermanshah, Iran

**Keywords:** Wound dressing, Nanofibers, *Momordica charantia*, Electrospinning, Collagen

## Abstract

**Background:**

Interactive dressings are innovatively designed to interact with the wound surface and alter the wound environment to promote wound healing. In the current study, we integrated the physicochemical properties of Poly (caprolactone)/ Poly (vinyl alcohol)/Collagen (PCL/PVA/Col) nanofibers with the biological activities of *Momordica charantia* pulp extract to develop an efficient wound dressing. The electrospinning method was applied to fabricate the nanofibers, and the prepared wound dressings were thoroughly characterized.

**Results:**

SEM imaging showed that the nanofibers were uniform, straight, without any beds with a diameter in the range of 260 to 480 nm. Increasing the concentration of the extract increased the diameter of the nanofibers and also the wettability characteristics while reduced the ultimate tensile strength from 4.37 ± 0.90 MPa for PCL/PVA/Col to 1.62 ± 0.50 MPa for PCL/PVA/Col/Ex 10% (*p* < 0.05). The in vivo studies showed that the application of the wound dressings significantly enhanced the healing process and the highest wound closure, 94.01 ± 8.12%, was obtained by PCL/PVA/Col/Ex 10% nanofibers (*p* < 0.05).

**Conclusion:**

The incorporation of the extract had no significant effects on nanofibers’ porosity, water vapor permeability, and swelling characteristics. The in vitro evaluations showed that the fabricated nanofibers were hemocompatible, cytocompatible, and prevent bacterial penetration through the dressing. These findings implied that the PCL/PVA/Col/Ex nanofibers can be applied as the wound dressing materials.

## Background

The skin has vital functions guaranteeing human health and its damages in any form must be appropriately treated. Wound healing is a multistep, complicated, and dynamic process involving various cells, biomolecules, and signaling pathways [1]. Wound healing/dressing materials have inevitable roles in promoting the healing process and protecting the process from interfering factors, such as microorganisms contamination, oxidative stress, and disrupting inflammatory responses [2]. Accordingly, unprecedented consideration must be given for developing efficient wound healing/dressing materials and structures [3]. Broadly speaking, wound dressings could be categorized as passive and active/interactive wound dressing. The first ones are passive protective structures with minimum biological activities favorable for wound healing. On the other hand, active/interfering wound dressings are designed to exhibit specific biological activity/ies beneficial for promoting the healing process and interfering with certain step/s of the healing process. Therefore, significant attention has been considered to develop efficient active/interactive wound dressings [4, 5].

Nanomaterials, with their numerous exceptional properties, have had revolutionary effects on various fields of biomedicine, as well as the wound care field. Using nanomaterials, it is possible to manipulate the biological processes more effectively and fabricate precisely tailored structures [6]. Nanofibers are fascinating structures with tremendous potential for skin and wound care applications due to their resemblance to the extracellular matrix (ECM) of skin, high surface to volume ratio, adjustable mechanical properties, porosity, and potential to load various drugs/bioactive molecules. Electrospun nanofibers are made from various natural, synthetic, and semi-synthetic polymers with different morphologies, architectures, and alignment [7–9]. Poly (caprolactone) (PCL) is a Food and Drug Administration (FDA)-approved synthetic polymer with a wide range of applications in biomedicine. Its biocompatibility and proper mechanical properties have grabbed significant attention for fabricating tissue engineering structures [10]. Poly (vinyl alcohol) (PVA) is another biocompatible FDA-approved synthetic polymers highly applicable in biomedical and pharmaceutical fields. Due to the solubility in water and facile formation of nanofibers, PVA is mostly applied as the carrier nanofibers [11–14].

Despite their proper mechanical properties and good processability, their applications in tissue engineering are restricted to apply as the passive structures. Accordingly, their combination with different biomolecules, biopolymers, and natural substances is an alternative approach to promotes their performances in regenerative medicine [15]. Collagen is a biopolymer with significant promising properties for biomedicine, especially skincare/regeneration applications. Collagen is the main ECM component in different organs with vital functions in tissue development and regeneration. It contains RGD (Arg-Gly-Asp) domains, which act as the cell attachment sites and enhance various tissue regenerative processes [16]. The combination of collagen with different synthetic structures was reported and resulted in excellent outcomes. In addition to biopolymers and biomolecules, natural substances such as plant extracts and gums could be used to promote the biological activities of synthetic polymers.

*Momordica charantia,* also known as bitter melon belongs to the Cucurbitaceae family, is cultivated in tropical and subtropical regions [17–19]. It is used in traditional medicines to treat microbial infections, inflammation, sluggish digestion, fever reduction, and wound-healing. Various biological activities have been reported for bitter melon such as anti-inflammatory, antioxidant, immunostimulatory, immunoregulatory, antidiabetic, hypocholesterolemic, hypoglycemic, cytoprotective, cardioprotective, antibacterial, antifungal, antiviral, and anticancer activities [20–24]. Hashmi et al. [25] fabricated *Momordica charantia*-loaded PVA nanofibers as the antibacterial structure. They reported that the prepared structure exhibited proper antibacterial activities activity against both gram positive and gram negative bacteria. In another study, Cui et al. [26] fabricated phlorotannin/*Momordica charantia* containing polyethylene oxide nanofibers as the active food packaging structure. They showed that the fabricated food packaging has antioxidant and antibacterial activities. Although there are some reports on the positive effects of bitter melon pulp extract on skin cells [27, 28], its application as a wound dressing component has not investigated. Accordingly, in the present study, we aimed to combine the favorable physical and biological properties of PCL/PVA/Col nanofibers with the biological activities of bitter melon pulp extract.

## Methods

### Materials collection

PCL (Mn = 70,000–90,000 gmol^− 1^), PVA (average molecular weight ~ 125,000 g mol^− 1^), and glutaraldehyde were purchased from Sigma-Aldrich (St. Louis MO, USA). Dimethyl sulfoxide (DMSO), chloroform, and glacial acetic acid were obtained from Merck (Darmstadt, Germany). MTT assay kit was purchased from Roth (Germany). Fetal Bovine Serum (FBS), DMEM/F-12 cell culture medium, Trypsin- EDTA, and Penicillin-Streptomycin (Pen-Strep) were obtained from Gibco (Germany). Xylazine and Ketamine were obtained from Alfasan (Netherlands). L929 murine fibroblastic cells and adult male Wistar rats were obtained from the Pasteur Institute, Iran.

### Extract preparation

In the extraction process, the pulp of *Momordica charantia* were air dried and chopped. Then, 40 g of the dried plant was soaked in 1 L of deionized (DI) water and heated for 10 min at 60 °C. The heated solution was passed through filter paper, and absolute ethanol was added to the filtered solution to precipitate the total extract. The resulted solution was passed again through the filter paper, collected the precipitate, frizzed for 12 h at − 20 °C, and freeze-dried at − 48 °C for 24 h using a freeze drier (Telstar, Terrassa, Spain) for 24 h at − 54 °C.

### Fabrication of nanofibrous wound dressing

A dual pump electrospinning system was used to fabricate nanofibrous wound dressing. PVA/Collagen nanofibers were fabricated as the carrier structure for the extract and PCL nanofibers for further strengthen the wound dressing. A proper amount of PCL was dissolved in chloroform for 12 h at ambient temperature to obtain 12 wt.% PCL solution. Another polymer solution containing 10 wt.% PVA and 10 wt.% collagen in acetic acid 0.02 N was prepared. Different concentrations of the extracts, 1, 5, and 10 wt.% respect the dried weight of polymers, were added to the prepared solution and stirred for 24 h at room temperature. The commercial dual pump electrospinning system (Fanavaran Nano Meghyas Ltd., Co., Tehran, Iran) comprises two syringe pumps on both sides of the rotating collector drum, which work independently was used. The PCL polymer solution was fed using one syringe pump and converted to nanofibers under the electrospinning parameters of feeding rate of 1 mL/min, applied voltage of 20 kV, and nozzle to collector distance of 10 cm. The PVA/Col/Ex polymer solution was fed using another syringe pump and converted to nanofibers using the same parameters. The fabricated nanofibers were cross-linked for biological experiments using vapor of 20% (v/v) glutaraldehyde at 37 °C for 6 h. The samples’ details were presented in Table 1 for convenience of the readers.

**Table 1:** The samples’ details of the prepared wound dressings

Samples’code	PCL concentration wt.%	PVA concentration wt.%	Collagen concentration wt.%	Extract concentration wt.%
S1	12	10	10	0
S2	12	10	10	1
S3	12	10	10	5
S4	12	10	10	10

### Characterization

#### Morphological observation

The morphology of the prepared nanofibers was observed using Scanning Electron Microscopy (SEM, Philips XL-30, Germany) at an accelerating voltage of 20 kV after sputter coating with a thin layer of gold using a sputter coater (SCD 004, Balzers, Germany). The diameter of the nanofibers was measured using Image J (1.47v, National Institute of Health, USA) software.

#### Wettability assessment

The prepared dressings’ wettability was assessed based on the sessile droplet contact angle method using a static contact angle measuring device (KRUSS, Hamburg, Germany).

#### Mechanical strength measurement

The mechanical properties of the prepared nanofibers were measured based on the tensile strength method according to the ISO 5270:1999 standard test methods using a uniaxial tensile testing device (Santam, Karaj, Iran) at a strain rate of 1 mm/min.

#### Swelling percent measurement

The prepared dressings’ swelling kinetics was evaluated based on the gravimetric measurement method in the phosphate-buffered saline solution (PBS, pH = 7.4) at ambient temperature. Briefly, a specific amount of nanofibers was weighed (W0), incubated in 20 mL PBS for 24, and weighed again (W1). Eq. 1 was used to calculate the water uptake value.

1 $$ \mathrm{Water}\ \mathrm{Uptake}\ \left(\%\right)=\frac{\mathrm{W}1-\mathrm{W}0}{\mathrm{W}0}\times 100 $$

#### Water vapor permeability (WVP) evaluation

The WVP is a determinant property of a wound dressing and evaluated based on our previous studies [29]. Mass of water evaporated from dressings-capped bottles through the dressing (W) for 12 h at 33 °C measured and applied in Eq. 2 to measure WVP.

2 $$ \mathrm{WVP}=\frac{\mathrm{W}}{\mathrm{AT}\ } $$

Where A is the area of dressing (1.18 cm^2^) and T is exposure time (12 h).

### Microbial penetration test

The resistance of a wound dressing against microbial penetration is a vital factor affecting a proper wound dressing’s efficiency. To assess this performance, the prepared dressings were capped 10 ml vials (test area: 0.8 cm2) containing BHI broth culture medium (5 mL) and growth of the bacteria into the culture medium was measured after 3 and 7 days. The positive control was an open vial, and the cotton ball-caped vial serves as the negative control group. The bacterial growth was assessed based on colony formation assay and measuring the culture medium’s turbidity, as the indication of bacterial growth, by spectroscopy approach at 600 nm using a Multi-Mode Microplate Reader (BioTek Synergy 2).

### Blood compatibility assay

Hemolysis induced by the prepared dressings was evaluated as an indication of hemocompatibility. 2 mL fresh and anticoagulated bold was diluted with 2.5 mL PBS and samples were incubated with 200 μL of the diluted blood for 60 min at 37 °C. After passing the incubation time, the samples were centrifuged for 10 min at 1500 rpm and the absorbance on the resulted supernatant was read at 545 nm using the Microplate Reader. Hemolysis percent was calculated using eq. 3.

3 $$ \mathrm{Hemolysis}\ \left(\%\right)=\frac{Dt- Dnc}{Dpc- Dnc}\times 100 $$

Where Dt is the absorbance of the sample, Dnc is the absorbance of the negative control, blood diluted with PBS whiteout any treatment, and Dpc is the absorbance of the positive control, blood lysed with DI.

### Cell culture studies

Biocompatibility of the prepared nanofibers against L929 murine fibroblastic cells was measured using an MTT assay kit at 24 and 72 cell seeding. Briefly, nanofibers were cut circulatory, put at the bottom of the 96-wells plate’s wells, sterilized by UV irradiation for 20 min, incubated with DMEM-F12 cells culture medium for 24 h, and thoroughly washed with sterile PBS. A number of 5000 cells in 100 μL cell culture medium supplemented with penicillin (100 unit/mL), streptomycin (100 μg/mL), and FBS (10% v/v). Cell-seeded nanofibers were incubated in a humidified incubator at 37 °C with 5% CO_2_ for 24 and 72 h. 100 μL of MTT solution (0.5 mg/mL) was added to each well after passing the incubation times and incubated for 4 h at 37 °C. Then, 100 μL DMSO was added to each well to dissolve the formed formazan crystals and the absorption of samples read at 570 nm using the Microplate Reader.

### Animal studies

The prepared nanofibers’ wound healing efficacy was evaluated based on the full-thickness model on 30 adult male Wistar rats (2 months old, weighing 200–220 g). The animal studies were approved by Kermanshah University of Medical Sciences (IR.KUMS.REC.1398.162) and conducted according to the National Institutes of Health guide for the care and use of Laboratory animals (NIH Publications No. 8023, revised 1978). Animals were randomly divided into 5 groups (6 rats per group), the negative control (without treatment), PCL/PVA/Col, PCL/PVA/Col/Ex 1%, PCL/PVA/Col/Ex 5%, PCL/PVA/Col/Ex 10%. A mixture of 70 mg/kg of Ketamine (5%) and 6 mg/kg of Xylazine 2% was i.p injected to induce general anesthesia, then, a circular full-thickness excisional wound with a diameter of 1 cm was induced on the back skin of rats. The nanofibrous wound dressings were fixed on the wound using elastic adhesive bandages.

### In vivo wound healing studies

The healing process was monitored by observing the macroscopic appearance of the wound and calculating the wound closure percent at different time points, as well as histopathological assessment at 14 days post-surgery. A digital camera (Canon Inc., Tokyo, Japan) was used to record the wound closure rate at 3, 7, 10, and 14 days post-treatment. The wound area was measured by an image analyzing program (Digimizer, Ostend, Belgium), and Eq. 4 was applied to calculate the wound closure percent.

4 $$ Wound\ closure\ \left(\%\right)=\left(1-\frac{Open\ wound\ area}{Initial\ wound\ area}\right)\times 100 $$

At 14 days post-treatment, the rats were sacrificed through the injection of high dose of anesthetic drugs (ketamine/Xylazine, 150/20 mg per kg). The wound area was extracted, fixed in paraformaldehyde (4% in PBS, 0.01 M, and pH 7.4), blocked, sectioned, and stained with Hematoxylin and Eosin (H&E) staining. An independent pathologist observed the specimens under a light microscope (Olympus, Tokyo).

### Statistical analysis

The statistical analysis was performed using the SPSS program, v.23 (IBM, Armonk, NY, USA) by applying a one-way ANOVA test with Tukey’s multiple comparison test (*p* < 0.05). All experiments were conducted in triplicate, except the animal studies performed on six rats in each group. The results were reported as a mean ± standard deviation, and *P* < 0.05 was considered statistically significant.

## Results and discussion

### Physical characterization results

#### Morphology observation

**Fig. 1:**
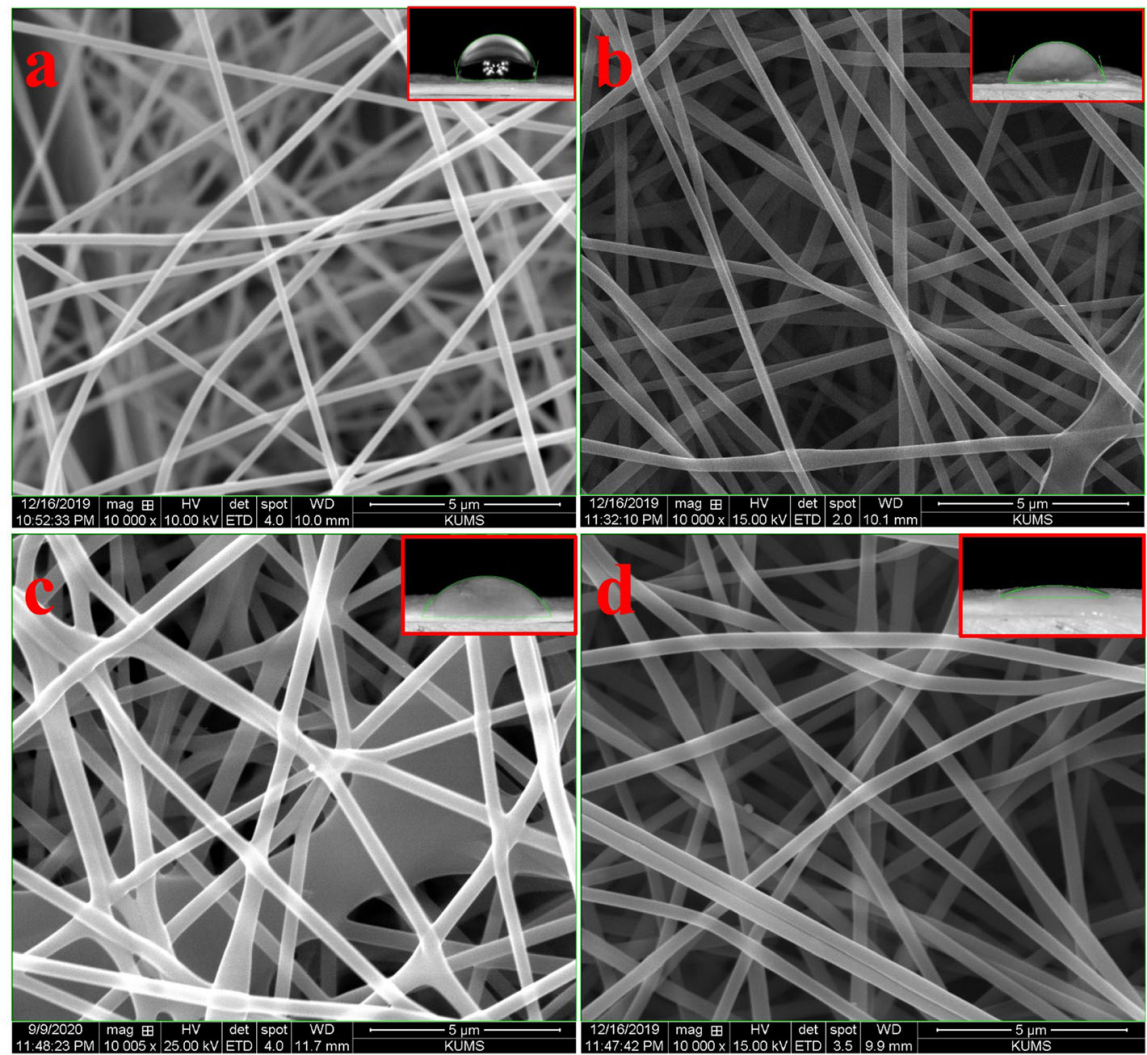
SEM micrograph of nanofibers. **a** S1, **b** S2, **c** S3, and **d** S4. Inserts are water contact results

The morphology of the prepared nanofibers was observed using SEM and the results showed that nanofibers were straight, uniform, and without any beads (Fig. 1). Increasing the concentration of extract had no adverse effects on the morphology of the nanofibers. The diameter of S1 was 284 ± 21 nm, and the addition of the extract increased the nanofibers’ diameter. The diameter of S2, S3, and S4 were 336 ± 50 nm, 394 ± 62 nm, and 430 ± 54 nm, respectively. This observation could be attributed to increasing the polymer solution’s viscosity under the addition of the extract. Zeyohanness et al. [30] reported that the addition of *Rhodomyrtus tomentosa* extract increased PVA nanofibers diameter. Suryamathi et al. [28] showed that *Tridax procumbens* extract’s immobilization on PCL nanofibers slightly increased the nanofibers’ diameter. Hashmi et al. [25] fabricated *Momordica charantia* loaded PVA nanofibers as the antibacterial structure. They reported that the addition of a high concentration of extract (50%) in PVA solution induced beads formation. They also reported that the increasing the concentration of the extract increased the nanofibers diameter.

#### Wettability results

The wettability of the prepared nanofibers was assessed based on the water contact angle measurement method and the results are presented in Table 2. The results showed that the WCA value of S1 was 99.11 ± 15.01° and the addition of the extract reduced the WCA. These results implied that the addition of the extract made the nanofibers more hydrophilic, which could be beneficial for wound healing applications due to the ability to absorb exudates and maintain the wound bed’s moisture [31].

**Table 2:** Characterization results of the prepared nanofibers

Samples	Contact angle (°)	UTS (MPa)	Porosity (%)	WVP mg/cm^2^/h	Water uptake ratio (%)
S1	99.11 ± 15.01	4.37 ± 0.90	80.07 ± 6.04	15.95 ± 1.21*	12. 21 ± 1.43
S2	74.76 ± 8.99	2.68 ± 0.05*	79.57 ± 8.26	15.17 ± 2.24*	15.32 ± 2.44
S3	64. 52 ± 11.04*	2.80 ± 0.85*	81.98 ± 5.26	16.01 ± 0.98*	15.98 ± 1.59
S4	22.92 ± 9.01*	1.62 ± 0.50*	75.31 ± 9.31	13. 90 ± 2.43*	20.93 ± 4.03*
Open container				31.03 ± 4.22	

Values represent the mean ± SD, *n* = 3. *p < 0.05 in comparison with the control group (obtained by one-way ANOVA)

#### Mechanical properties

**Fig. 2:**
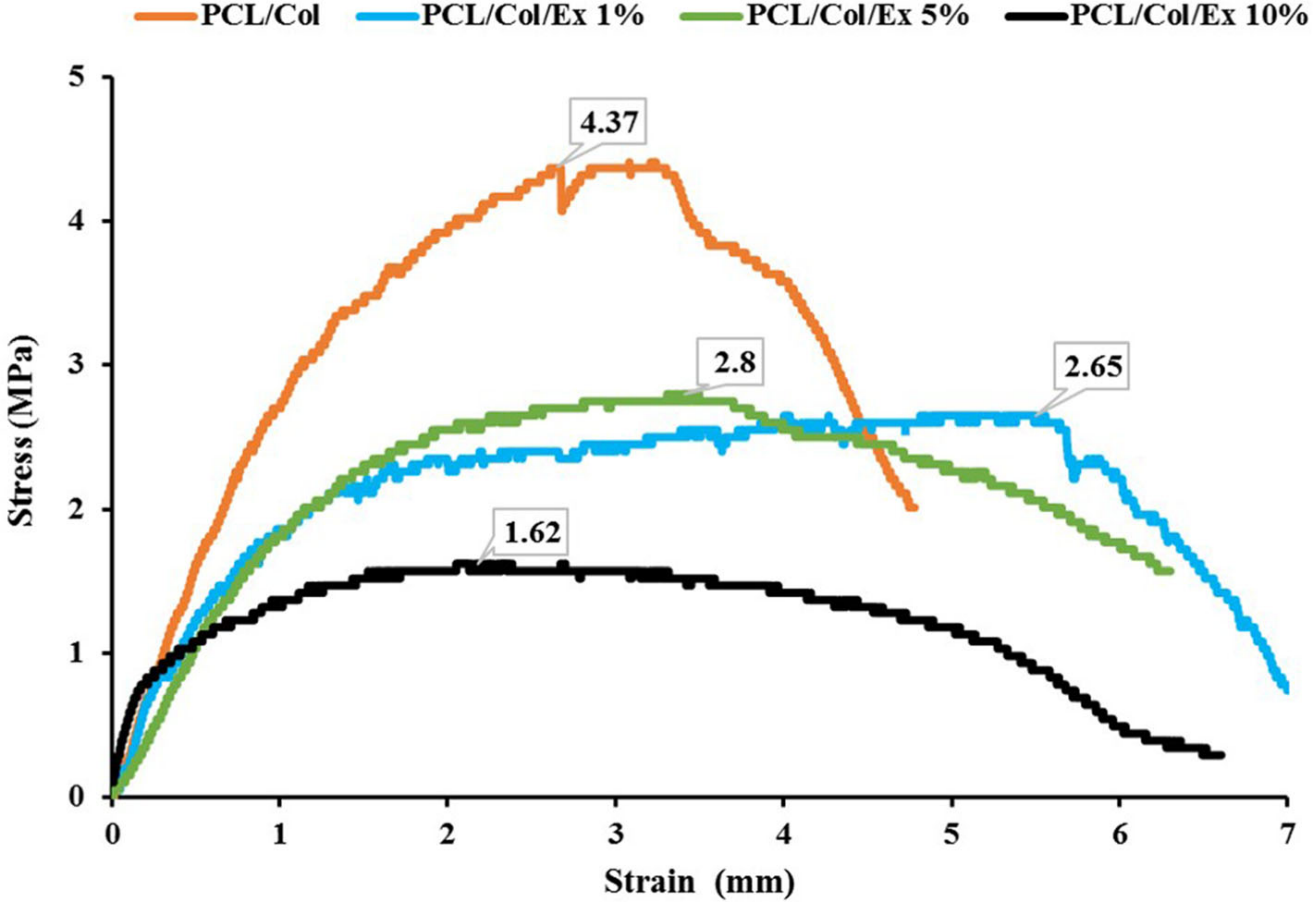
Stress–strain curves and tensile testing results of the prepared nanofibers during tensile loading

The mechanical properties of the prepared nanofibers were evaluated based on the tensile strength method and the results are presented in Table 2 and Fig. 2. The results showed that the ultimate tensile strength (UTS) value for S1 was 4.37 ± 0.90 MPa and significantly reduced with the addition of the extract (*P* < 0.05). In our previous study, we observed that the mechanical strength of cellulose acetate/gelatin nanofiber compromised with the addition of berberine [29]. This may be due to the weakening of the interactions between polymers chains by incorporating the extract. It is reported that the Waals forces and hydrogen bonding between polymer chains could be affected by the addition of natural substance [32]. Moreover, it was observed that the elasticity of the nanofibers was increased with the addition of extract. Hashmi et al. [25] showed that the incorporation of *Momordica charantia* extract decreased the mechanical strength of neat PVA nanofibers. They proposed that the observed compromised mechanical strength could be related to the adverse effect of the extract on the crystallinity of PVA. The obtained results in the present study indicated that the prepared dressings’ mechanical properties are beneficial for wound healing/dressing applications. It is proposed that the tensile strength of 0.7 to 18.0 MPa is sufficient for skin tissue engineering/care applications [33, 34].

#### Porosity values

The porosity of the prepared wound dressings was measured based on the liquid displacement method and the results are presented in Table 2. The results showed that the porosity of the nanofibers was in the range of 70 to 85%. Moreover, it was observed that the addition and concentration of the extract did not significantly affect the porosity (*P* < 0.3). Furthermore, it is reported that the porosity between 60 to 90% is suitable for tissue engineering applications, supporting cells infiltration, and nutrition/waste transition [35].

#### Swelling results

The swelling kinetics of the prepared nanofibers was measured by the gravimetric method, and the obtained results are presented in Table 2. The results showed that the water uptake vale of S1 was 12.21 ± 1.43% and increased to15.32 ± 2.44, 15.98 ± 1.59, and 20.93 ± 4.03% with the addition of 1, 5, and 10% extract, respectively. The observed increased water uptake values can be related to increasing the nanofibers’ hydrophilicity with increasing the extract. According to the obtained results, it can be concluded that the prepared dressings are able to absorb wound exudates.

#### Water vapor permeability potential

According to the moist wound healing hypothesis, the epithelialization occurs twice the rate in the moist wound bed compared to a dry one. The wound bed’s moisture is directly related to the water vapor permeability and swelling characteristics of the wound dressing [36]. The water vapor permeability rate of the prepared nanofibers was measured and the results are presented in Table 2. The results showed that the application of the nanofibers halved the permeability rate compared to the negative control (open container) (*p* < 0.05). It was observed that the addition and concentration of the extract had no significant effect on the vapor permeability (*p* < 0.2).

### Biological evaluations

#### Microbial evaluations findings

Protecting the wound area from bacterial invasion is another critical function of a proper wound dressing. The results showed that the application of the prepared nanofibrous dressings significantly hindered bacterial penetration through the nanofibers (*p* < 0.05) (Table 3). It is reported that the application of even multi-layers of gauze cannot protect the wound bed from bacterial penetration [37]. These findings depict that the prepared nanofibrous dressings are suitable to manage the situations of the wound bed.

**Table 3:** Microbial barrier property of the fabricated dressing after 3 and 7 days of incubation, measured by colony counting assay and spectroscopy method

Samples	Microbial barrier properties			
	Number of colonies		Absorbance at 600 nm (OD)	
	***3 days***	***7 days***	***3 days***	***7 days***
Positive Control	80.11 ± 12.10	240.89 ± 24.11	6.01 ± 1.5	14.31 ± 3.20
Negative Control	1.24 ± 0.55	1.82 ± 0.45	0.48 ± 0.01	0.59 ± 0.21
S1	1.52 ± 0.63*	1.90 ± 0.80*	0.86 ± 0.09*	1.25 ± 0.78*
S2	1.50 ± 0.26*	1.77 ± 0.39*	0.56 ± 0.036*	6.98 ± 0.039*
S3	1.77 ± 0.81*	2.01 ± 0.89*	0.95 ± 0.059*	1.20 ± 0.09*
S4	1.49 ± 0.19*	1.65 ± 0.90*	0.51 ± 0.075 *	0.60 ± 0.083*

Values represent the mean ± SD, *n* = 3. *p < 0.05 in comparison with the positive control group (obtained by one-way ANOVA)

#### Blood compatibility findings

**Fig. 3:**
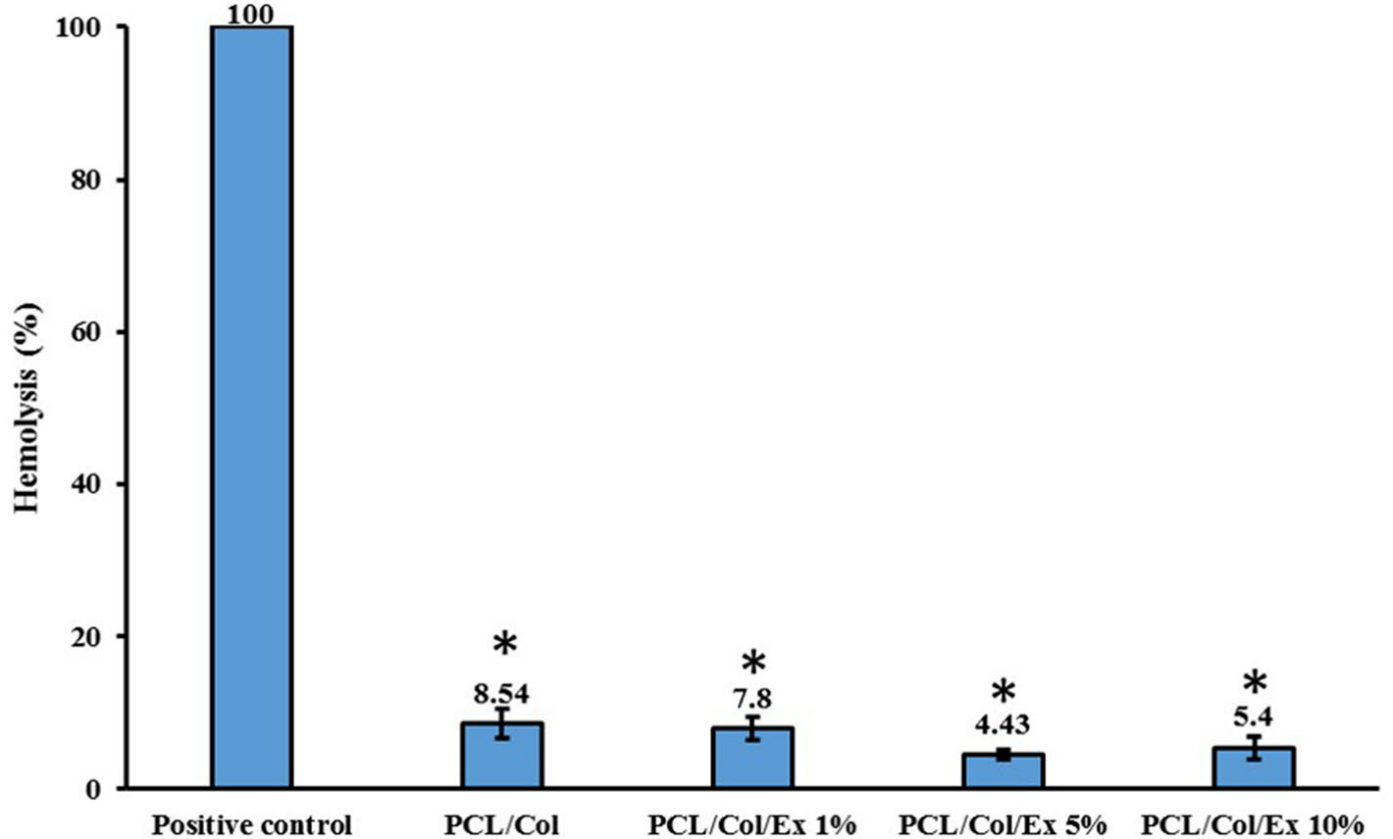
The hemolysis percent induced by the prepared nanofibers. The positive control was blood treated with water. Values represent the mean ± SD, *n* = 3, *p < 0.05 (obtained by one-way ANOVA)

Blood compatibility is a property that should be checked for any biomaterials dedicated to being in contact with blood elements. In this study, possible hemolysis induced by the prepared nanofibrous wound dressing materials was evaluated as the indication of blood compatibility. As shown in Fig. 3, the nanofibers did not cause significant hemolysis, and the observed hemolysis was significantly lower than the positive control. Moreover, it was observed that increasing the concentration of the extract slightly reduced the hemolysis, which was not statistically significant compared with S1 group (*p* < 0.2).

#### In vitro toxicity result

**Fig. 4:**
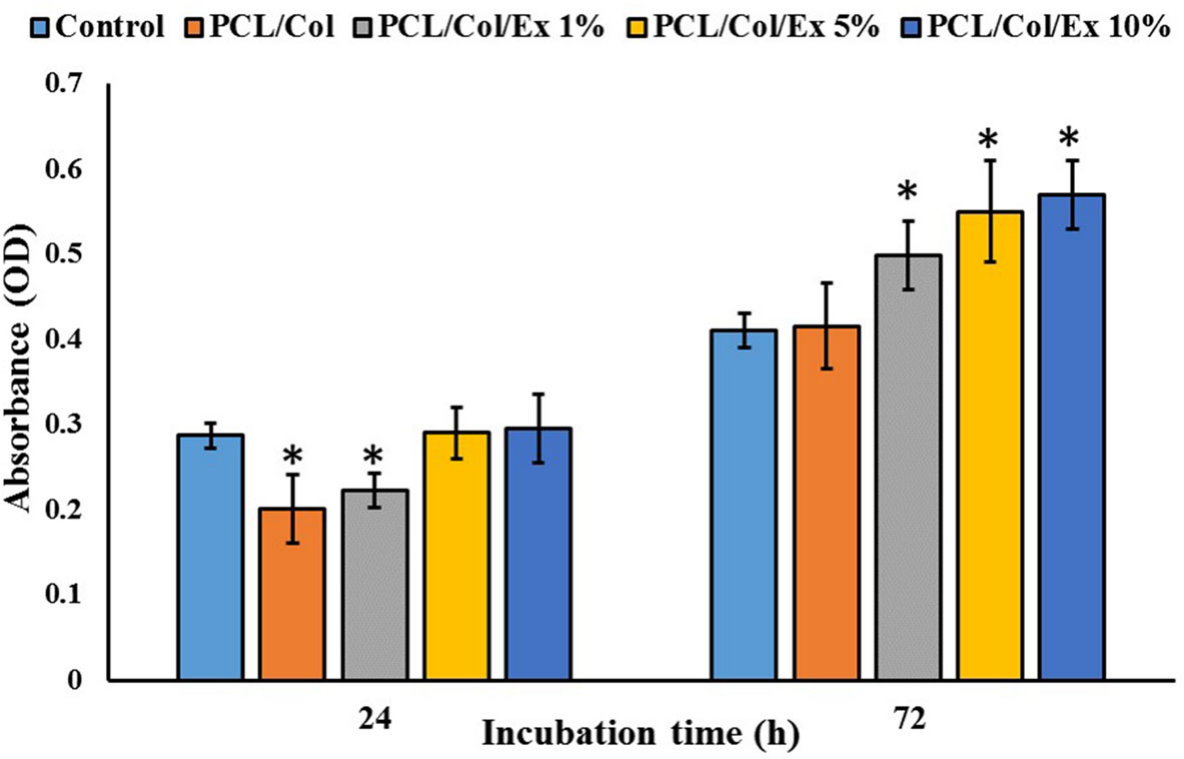
Viability of L929 murine fibroblastic cell on the prepared nanofibers measured by MTT assay at 24and 72 h post-cell seeding. Control: Tissue Culture Plastic (TCP). Values represent the mean ± SD, *n* = 5, *p < 0.05 (obtained by one-way ANOVA)

The in vitro toxicity of the prepared nanofibers was measured using a MTT assay kit at 24 and 72 h (Fig. 4). The results showed that cells viability on the S1 and S2 was lower than the control group (*p* < 0.05) and the cell viability on the S3 and S4 were equal to the control. This can be related to the low adaptation of cells with nanofibers at 24 h. On the other hand, cell growth on the extract containing nanofibers was significantly increased with passing time (*p* < 0.05). These findings implied that the fabricated nanofibers are suitable for cell growth. Moreover, incorporating the extract significantly increased the cell proliferation and the highest growth was observed in the S4 group. Hashmi et al. [25] showed that electrospun *Momordica charantia* incorporated PVA nanofibers supported the fibroblast cells (NIH3T3) and exhibited proper biocompatibility. The positive effects of bitter melon pulp extract on cell proliferation are reported in previous studies. Ono et al. [38] reported that bitter melon pulp extract stimulates human dermal fibroblasts proliferation through the indirect activation of mitogen-activated protein kinases (MAPKs), especially the ERK pathway. Their molecular investigations revealed enhanced [3H] thymidine incorporation under treatment with the extract. They proposed that the extract can be useful for tissue regeneration applications, especially wound healing proposes. In another study, Park et al. [27] showed that *Momordica charantia* extract promotes the proliferation of HaCaT keratinocytes.

#### In vivo wound-healing outcomes

**Fig. 5:**
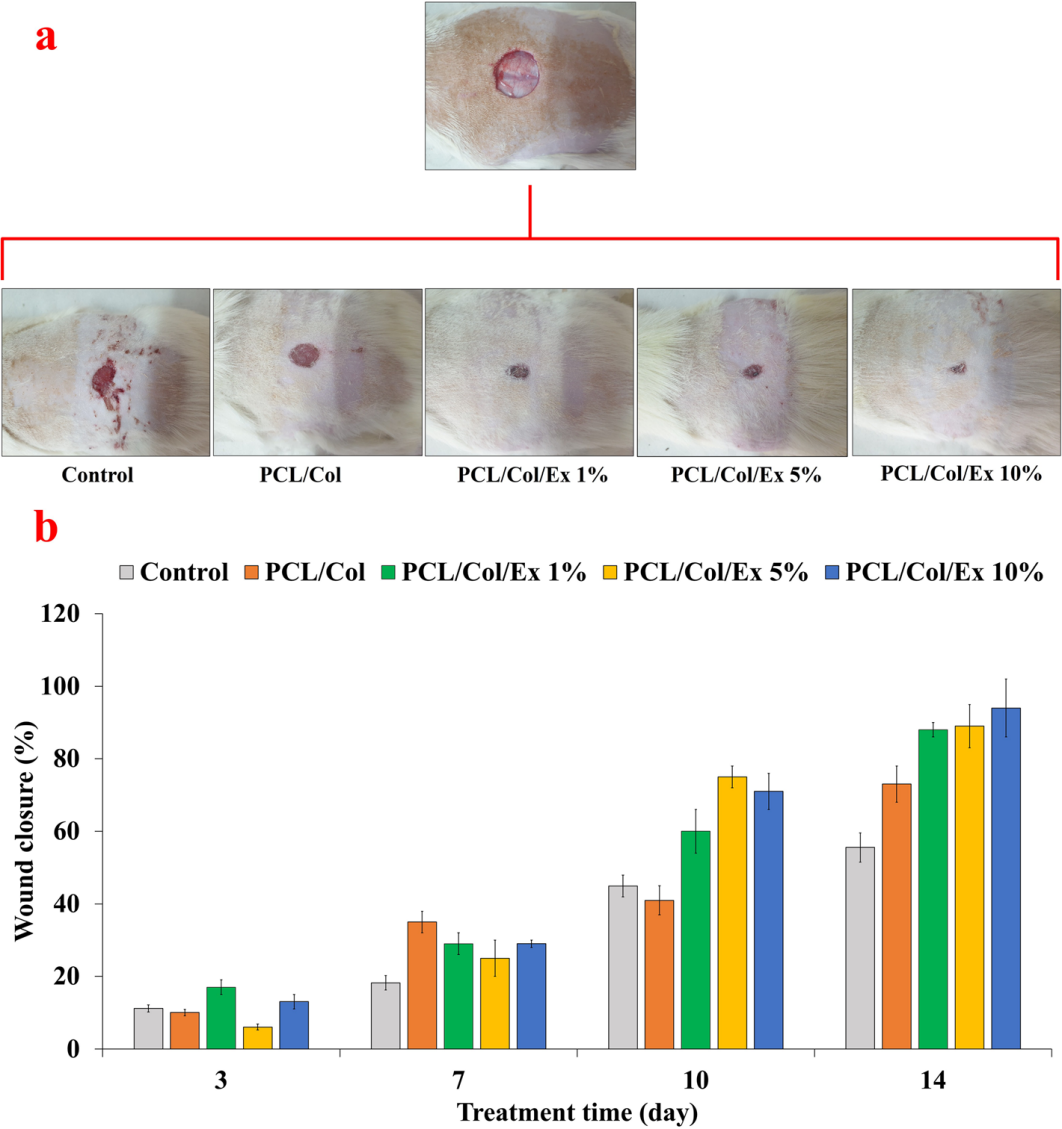
In vivo wound-healing results: **a** macroscopic appearances of wounds treated 14 days post-treatment, **b** Histogram comparing the wound closure at different post-treatment days. Values represent the mean ± SD, *n* = 6, *p < 0.05

**Fig. 6:**
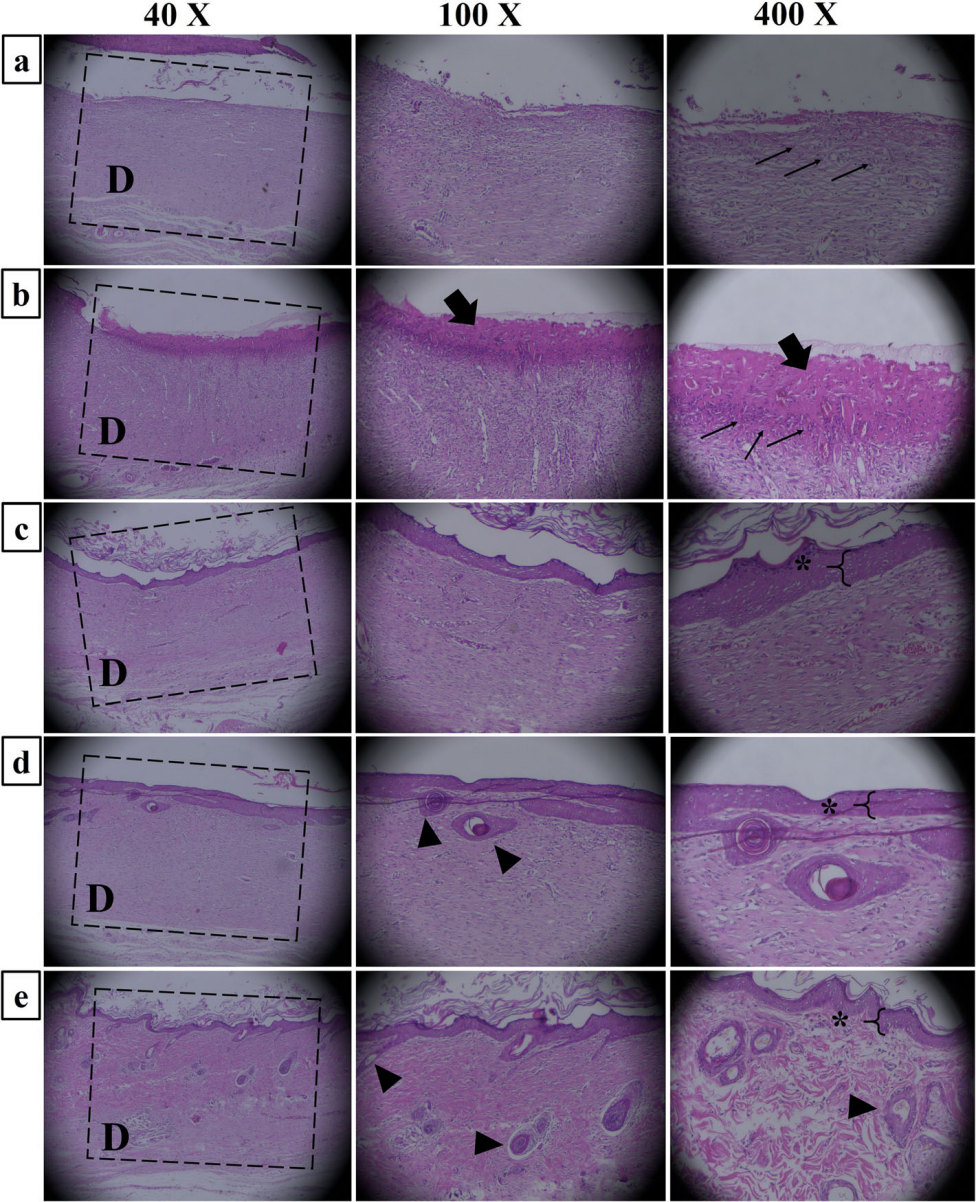
Hematoxylin and eosin (H&E) stained microscopic sections of healed incisions in rats at 14 days. **a** Negative control, **b** S1, **c** S2, **d** S3, and **e** S4. D: defect, Thin arrows: infiltration of inflammatory cells, Thick arrows: crusty scab, Star: epid

The wound healing performance of the prepared nanofibers was evaluated in the full-thickness animal model drug 14 days. The assessments were conducted via macroscopic, the wound closure (Fig. 5), and microscopic, histological observations (Fig. 6). The wound closure assessment results showed that the induced wound was closed under treatment with extract-incorporated dressing for 14 days. Moreover, the highest wound closure value was obtained under treatment with PCL/PVA/Col/Ex 10% with the value of 94.01 ± 8.12%, which was statistically significant than the negative control groups, 55.54 ± 5.32 (*p* < 0.05).

The histopathological observation was conducted for a detailed evaluation of the healing process, and the results are presented in Fig. 6. with different magnifications. The results showed that in the negative control group, the epidermal layer has not formed yet and the infiltration of the inflammatory cells is dominant (Fig. 6, thin arrows). In the PCL/PVA/Col group, the wound was covered with a crusty scab and epidermal tissue formation is not completed. Moreover, the infiltration of inflammatory cells is seen. In the groups treated with extract-incorporated dressings, healing signs are more dominant compared with the negative control group and PCL/PVA/Col group. In PCL/PVA/Col/Ex 1% group, the epidermal layer is partially formed, and the population of inflammatory cells is significantly decreased. In PCL/PVA/Col/Ex 5% and PCL/PVA/Col/Ex 10% groups, the epidermal layer is formed and signs of the hair follicle and sebaceous gland were seen in this group. These results implied that the application of bitter melon extract-incorporated nanofibers can accelerate the wound healing process.

The effects of better melon pulp extract on skin regenerating cells are reported by various researchers. Park et al. [27] showed that *Momordica charantia* extract exhibited antioxidant and cytoprotective activities on HaCaT keratinocytes and H2O2-damaged HaCaT cells through the activation or expression induction of HO-1, AKT, Kelch-like ECH-associated protein 1 (KEAP1), and p85/PI3K. They also proposed that the extract treatment can regulate the expression or activity of tissue remodeling factors such as matrix metalloproteinase (MMP)-1 and − 9, elastase, and type 1 collagen, as well as tissue-protecting enzymes such as sirtuin 1 (SIRT1) and hemeoxygenase-1 (HO-1) and in HaCaT and NIH3T3 fibroblasts cells. In another study, Kumar et al. [39] reported the antioxidant and cytoprotective effects of the extract on xanthin oxidase (HX − XO) and H2O2-damaged keratinocyte (A431), NIH 3 T3, and rat cardiac fibroblasts (RCFs) cells. These studies proposed that better melon pulp extract’s main wound healing activities are due to its radical scavenging, antioxidant, and cytoprotective properties.

## Conclusion

The combination of nanofibers with natural substances with positive biological activities is a promising approach to develop bioactive/interactive wound dressing materials. Nanofibers offer fascinating properties and performance such as a high surface to volume ratio suitable for drug delivery applications and adjustable porosity beneficial for controlling the water vapor and oxygen exchange between the wound bed and surrounding environment. Various studies reported the positive effects of better melon pulp extract on skin cells. Accordingly, in this study, we aimed to combine PCL/PVA/Col nanofibers’ physical and biological properties with bitter melon pulp extract’ antioxidant, cytoprotective, and proliferative activities. The obtained results showed that the prepared nanofibers possess acceptable physical properties such as nanofibers diameter, wettability, porosity, mechanical properties, swelling kinetic, water vapor permeability, and bacterial penetration barrier. These performances provide a proper environment for wound healing.

The incorporation of bitter melon (*Momordica charantia*) pulp extract confer beneficial biological properties to the prepared nanofibers. The nanofibers were hemocompatible and cytocompatible, confirmed with the in vitro studies. Moreover, the in vivo studies revealed that the prepared nanofibers could promote the wound healing process. These findings indicate that the prepared nanofibers could be effective as the wound dressing materials. Although further studies are required to reveal the exact healing mechanism of the fabricated wound dressing. Moreover, due to the better melon extract’s antidiabetic effects, the prepared wound dressing could be considered and evaluated for diabetic wound healing applications.

## Data Availability

The datasets used and/or analyzed during the current study are available from the corresponding author on reasonable request.

## References

[1] Monfared GS, Ertl P, Rothbauer M (2020). An on-chip wound healing assay fabricated by xurography for evaluation of dermal fibroblast cell migration and wound closure. Sci Rep.

[2] Eskandarinia A, Kefayat A, Agheb M, Rafienia M, Baghbadorani MA, Navid S, Ebrahimpour K, Khodabakhshi D, Ghahremani F (2020). A novel bilayer wound dressing composed of a dense polyurethane/Propolis membrane and a biodegradable Polycaprolactone/gelatin Nanofibrous scaffold. Sci Rep.

[3] Ullah A, Ullah S, Khan MQ, Hashmi M, Nam PD, Kato Y, Tamada Y, Kim IS (2020). Manuka honey incorporated cellulose acetate nanofibrous mats: fabrication and in vitro evaluation as a potential wound dressing. Int J Biol Macromol.

[4] Ambekar RS, Kandasubramanian B (2019). Advancements in nanofibers for wound dressing: a review. Eur Polym J.

[5] Obagi Z, Damiani G, Grada A, Falanga V. Principles of wound dressings: a review. Surgical technology international. 2019;35:50–7. 31480092

[6] Soleimani K, Arkan E, Derakhshankhah H, Haghshenas B, Jahanban-Esfahlan R, Jaymand M (2020). A novel bioreducible and pH-responsive magnetic nanohydrogel based on β-cyclodextrin for chemo/hyperthermia therapy of cancer. Carbohydr Polym.

[7] Samadian H, Mobasheri H, Hasanpour S, Ai J, Azamie M, Faridi-Majidi R (2020). Electro-conductive carbon nanofibers as the promising interfacial biomaterials for bone tissue engineering. J Mol Liq.

[8] Ullah S, Ullah A, Lee J, Jeong Y, Hashmi M, Zhu C, Joo KI, Cha HJ, Kim IS (2020). Reusability comparison of melt-blown vs nanofiber face mask filters for use in the coronavirus pandemic. ACS Applied Nano Materials.

[9] Hashmi M, Ullah S, Kim IS (2019). Copper oxide (CuO) loaded polyacrylonitrile (PAN) nanofiber membranes for antimicrobial breath mask applications. Curr Res Biotechnol.

[10] Fazlalizadeh F, Massoumi B, Banaei A, Jaymand M. A thermal-responsive Y-shaped Miktoarm Amphiphilic block copolymer composed of poly (ε-caprolactone) and poly (N-isopropylacrylamide) as a Nano-micellar carrier for anti-cancer drugs. Polym Sci Ser B. 2020;62:1–10.

[11] Massoumi B, Jafarpour P, Jaymand M, Entezami AA (2015). Functionalized multiwalled carbon nanotubes as reinforcing agents for poly (vinyl alcohol) and poly (vinyl alcohol)/starch nanocomposites: synthesis, characterization and properties. Polym Int.

[12] Hashmi M, Ullah S, Ullah A, Khan MQ, Hussain N, Khatri M, Bie X, Lee J, Kim IS (2020). An optimistic approach “from hydrophobic to super hydrophilic nanofibers” for enhanced absorption properties. Polym Test.

[13] Hashmi M, Ullah S, Ullah A, Akmal M, Saito Y, Hussain N, Ren X, Kim IS (2020). Optimized loading of carboxymethyl cellulose (CMC) in tri-component electrospun nanofibers having uniform morphology. Polymers.

[14] Ullah S, Hashmi M, Hussain N, Ullah A, Sarwar MN, Saito Y, Kim SH, Kim IS (2020). Stabilized nanofibers of polyvinyl alcohol (PVA) crosslinked by unique method for efficient removal of heavy metal ions. J Water Process Eng.

[15] Samadian H, Maleki H, Allahyari Z, Jaymand M (2020). Natural polymers-based light-induced hydrogels: promising biomaterials for biomedical applications. Coord Chem Rev.

[16] Samadian H, Maleki H, Fathollahi A, Salehi M, Gholizadeh S, Derakhshankhah H, Allahyari Z, Jaymand M (2020). Naturally occurring biological macromolecules-based hydrogels: potential biomaterials for peripheral nerve regeneration. Int J Biol Macromol.

[17] Khan MF, Abutaha N, Nasr FA, Alqahtani AS, Noman OM, Wadaan MA (2019). Bitter gourd (Momordica charantia) possess developmental toxicity as revealed by screening the seeds and fruit extracts in zebrafish embryos. BMC Complement Altern Med.

[18] Sagkan RI (2013). An in vitro study on the risk of non-allergic type-I like hypersensitivity to Momordica charantia. BMC Complement Altern Med.

[19] Fernandes NP, Lagishetty CV, Panda VS, Naik SR (2007). An experimental evaluation of the antidiabetic and antilipidemic properties of a standardized Momordica charantia fruit extract. BMC Complement Altern Med.

[20] Grover J, Yadav S (2004). Pharmacological actions and potential uses of Momordica charantia: a review. J Ethnopharmacol.

[21] Panda BC, Mondal S, Devi KSP, Maiti TK, Khatua S, Acharya K, Islam SS (2015). Pectic polysaccharide from the green fruits of Momordica charantia (Karela): structural characterization and study of immunoenhancing and antioxidant properties. Carbohydr Res.

[22] Raish M (2017). Momordica charantia polysaccharides ameliorate oxidative stress, hyperlipidemia, inflammation, and apoptosis during myocardial infarction by inhibiting the NF-κB signaling pathway. Int J Biol Macromol.

[23] Raish M, Ahmad A, Jan BL, Alkharfy KM, Ansari MA, Mohsin K, al Jenoobi F, Al-Mohizea A (2016). Momordica charantia polysaccharides mitigate the progression of STZ induced diabetic nephropathy in rats. Int J Biol Macromol.

[24] Chaturvedi P (2012). Antidiabetic potentials of Momordica charantia: multiple mechanisms behind the effects. J Med Food.

[25] Hashmi M, Ullah S, Kim IS (2020). Electrospun Momordica charantia incorporated polyvinyl alcohol (PVA) nanofibers for antibacterial applications. Mater Today Commun.

[26] Cui H, Yang X, Abdel-Samie MA, Lin L (2020). Cold plasma treated phlorotannin/Momordica charantia polysaccharide nanofiber for active food packaging. Carbohydr Polym.

[27] Park SH, Yi Y-S, Kim M-Y, Cho JY (2019). Antioxidative and antimelanogenesis effect of momordica charantia methanol extract. Evid Based Complement Alternat Med.

[28] Suryamathi M, Ruba C, Viswanathamurthi P, Balasubramanian V, Perumal P (2019). Tridax procumbens extract loaded electrospun PCL nanofibers: a novel wound dressing material. Macromol Res.

[29] Samadian H, Zamiri S, Ehterami A, Farzamfar S, Vaez A, Khastar H, et al. Electrospun cellulose acetate/gelatin nanofibrous wound dressing containing berberine for diabetic foot ulcer healing: in vitro and in vivo studies. Sci Rep. 2020;10(1). 10.1038/s41598-020-65268-7. 10.1038/s41598-020-65268-7 PMC7239895 32433566

[30] Zeyohanness SS, Abd Hamid H, Zulkifli FH (2018). Poly (vinyl alcohol) electrospun nanofibers containing antimicrobial Rhodomyrtus tomentosa extract. J Bioact Compat Polym.

[31] Jin SG, Yousaf AM, Kim KS, Kim DW, Kim DS, Kim JK, Yong CS, Youn YS, Kim JO, Choi H-G (2016). Influence of hydrophilic polymers on functional properties and wound healing efficacy of hydrocolloid based wound dressings. Int J Pharm.

[32] Samadian H, Ehterami A, Sarrafzadeh A, Khastar H, Nikbakht M, Rezaei A, Chegini L, Salehi M (2020). Sophisticated polycaprolactone/gelatin nanofibrous nerve guided conduit containing platelet-rich plasma and citicoline for peripheral nerve regeneration: in vitro and in vivo study. Int J Biol Macromol.

[33] Barnes CP, Sell SA, Boland ED, Simpson DG, Bowlin GL (2007). Nanofiber technology: designing the next generation of tissue engineering scaffolds. Adv Drug Deliv Rev.

[34] Nseir N, Regev O, Kaully T, Blumenthal J, Levenberg S, Zussman E (2013). Biodegradable scaffold fabricated of electrospun albumin fibers: mechanical and biological characterization. Tissue Engineering Part C Methods.

[35] Chong EJ, Phan TT, Lim IJ, Zhang Y, Bay BH, Ramakrishna S, Lim CT (2007). Evaluation of electrospun PCL/gelatin nanofibrous scaffold for wound healing and layered dermal reconstitution. Acta Biomater.

[36] Xu R, Xia H, He W, Li Z, Zhao J, Liu B, Wang Y, Lei Q, Kong Y, Bai Y (2016). Controlled water vapor transmission rate promotes wound-healing via wound re-epithelialization and contraction enhancement. Sci Rep.

[37] Babu M (2000). Collagen based dressings—a review. Burns.

[38] Ono T, Tsuji T, Sakai M, Yukizaki C, Ino H, Akagi I, Hiramatsu K, Matsumoto Y, Sugiura Y, Uto H (2009). Induction of hepatocyte growth factor production in human dermal fibroblasts and their proliferation by the extract of bitter melon pulp. Cytokine.

[39] Kumar R, Balaji S, Sripriya R, Nithya N, Uma T, Sehgal P (2010). In vitro evaluation of antioxidants of fruit extract of Momordica charantia L. on fibroblasts and keratinocytes. J Agric Food Chem.

